# Histologic patterns of liver injury induced by anti-PD-1 therapy

**DOI:** 10.1093/gastro/goz044

**Published:** 2019-09-20

**Authors:** Dongwei Zhang, John Hart, Xianzhong Ding, Xuchen Zhang, Michael Feely, Lindsay Yassan, Lindsay Alpert, Consuelo Soldevila-Pico, Xuefeng Zhang, Xiuli Liu, Jinping Lai

**Affiliations:** 1 Department of Pathology, Immunology and Laboratory of Medicine, University of Florida College of Medicine, Gainesville, FL, USA; 2 Department of Pathology and Laboratory Medicine, University of Rochester Medical Center, Rochester, NY, USA; 3 Department of Pathology, University of Chicago Pritzker School of Medicine, Chicago, IL, USA; 4 Department of Pathology and Laboratory of Medicine, Loyola University Medical Center, Maywood, IL, USA; 5 Department of Pathology, Yale School of Medicine, New Haven, CT, USA; 6 Department of Medicine, University of Florida College of Medicine, Gainesville, FL, USA; 7 Department of Pathology, Duke University Medical Center, Durham, NC, USA; 8 Department of Pathology, Kaiser Permanente Sacramento Medical Center, Sacramento, CA, USA

**Keywords:** nivolumab, pembrolizumab, anti-PD-1, liver injury, histology, hepatitis

## Abstract

**Background:**

Nivolumab and pembrolizumab—two monoclonal antibodies that block human programmed cell death-1 (PD-1)—have been successfully used to treat patients with multiple advanced malignancies. The histologic patterns of hepatic toxicity induced by anti-PD-1 treatment have not been well studied and the aim of this study was to explore them.

**Methods:**

Eight patients with advanced malignancies who were treated with either nivolumab or pembrolizumab were identified from five institutions. These patients had no history of underlying liver disease and a viral hepatitis panel was negative in all patients.

**Results:**

Seven of eight patients exhibited mild to moderate gastrointestinal symptoms such as abdominal pain, fatigue, nausea, vomiting, and jaundice after anti-PD-1 treatment. Significant elevations in liver-chemistry tests were detected in all patients. Six cases (6/8) demonstrated an acute lobular hepatitis pattern of histologic injury. The remaining two cases showed different histologic patterns of injury: steatohepatitis with mild cholestasis (1/8) and pure acute cholestatic injury (1/8). No case showed typical features of autoimmune hepatitis. The liver function recovered in all eight cases after cessation of anti-PD-1 agents and with immunosuppressive therapy.

**Conclusions:**

Our study suggests that screening patients for abnormal liver-function tests prior to anti-PD-1 therapy as well as periodic monitoring of liver-function tests are necessary to prevent severe liver injury. Rather than causing classical autoimmune hepatitis, PD-1 inhibitors appear to produce an immune-mediated nonspecific acute hepatitis. Drug cessation, without steroid therapy, may therefore be sufficient in some patients.

## Introduction

Targeted immunotherapy as a potential treatment for cancer has been intensively studied over the past decade [1, 2]. Tumor cells often use multiple resistance mechanisms to evade the host immune system [3, 4]. Checkpoint proteins, such as programmed cell death-1 (PD-1) on T lymphocytes and PD-1 ligand (PD-L1) receptors on tumor cells, allow tumor cells to keep the host immune response in check [5, 6]. PD-1 is an inhibitory receptor expressed on activated T and B cells that limits the activity of T cells at a variety of stages of the immune response [7, 8]. Blocking the binding of PD-L1 to PD-1 with an immune checkpoint inhibitor (anti-PD-L1 or anti-PD-1 antibodies) can enhance antitumor responses and delay tumor growth [9, 10]. These antibodies have shown promising results in treating several types of malignancies in recent years [2].

Nivolumab (Opdivo) and pembrolizumab (Keytruda)—two human immunoglobulin G4 (IgG4) monoclonal antibodies targeting PD-1—are now used to treat patients with advanced tumors such as metastatic melanoma, non-small-cell lung cancer, squamous-cell carcinoma of the head and neck, urothelial carcinoma, Hodgkin lymphoma, and mismatch repair-deficient (dMMR) solid tumormismatch repair-deficient (dMMR) solid tumors [11–18]. Unfortunately, the use of these drugs can cause activating the immune system to attack normal organs, leading to a unique pattern of autoimmune-like/inflammatory side effects in some patients [19–21]. For example, liver-function abnormalities, such as increased alanine transaminase (ALT), aspartate transaminase (AST), alkaline phosphatase, and bilirubin, occurred in ≥10% of nivolumab-treated patients and at a higher occurrence rate than that in chemotherapy-treated patients [11, 16, 22]. The hepatitis induced by anti-PD-1 therapy has been reported as single case reports or small series [23, 24]. However, the hepatic histopathologic pattern of liver toxicity in these patients has not been well studied. This study aims to identify the histologic patterns of hepatic injury induced by anti-PD-1 treatment.

## Patients and methods

Eight patients with advanced malignancies who were treated with nivolumab or pembrolizumab between 2016 and 2018 from the University of Florida College of Medicine (*n *=* *2), University of Chicago Pritzker School of Medicine (*n *=* *3), Loyola University Medical Center (*n *=* *1), Yale School of Medicine (*n *=* *1), and Duke University Medical Center (*n *=* *1) were included in this study. Data on clinical presentation, medical history, laboratory tests, imaging, treatment, and patient outcomes were recorded. The patients did not have any previous history of underlying liver disease, except mild steatosis due to obesity in one patient. Liver-function tests were normal before treatment and viral hepatitis panels were negative before and after treatment. Liver biopsies were performed to evaluate the pattern of hepatic toxicity.

## Results

### Clinical characteristics

Clinical data are summarized in Table 1. Six patients were male and two were female. The mean age was 55 years (range, 42–69 years). Primary cancers included three metastatic melanomas, one metastatic pancreatic adenocarcinoma, one metastatic squamous-cell carcinoma of the head and neck, one ovarian high-grade serous carcinoma, one refractory Hodgkin lymphoma, and one glioblastoma. Six patients received a mean of three (range, 1–8) cycles of nivolumab and two patients received a mean of five (range, 4–6) cycles of pembrolizumab before liver biopsy. Seven out of eight patients exhibited mild to moderate gastrointestinal symptoms such as abdominal pain, fatigue, nausea, vomiting, and jaundice. Liver-function abnormalities such as elevated ALT, AST, and alkaline phosphatase were detected in all of the cases. ALT and AST were above five times the upper limit of normal in six out of eight patients. Elevated total bilirubin was detected in three cases. Imaging studies were performed in two patients and showed no biliary obstruction or intrahepatic dilatation.

**Table 1.** Clinical characteristics of eight patients with anti-PD-1 treatment

Pt#	Age/gender	Malignancy	Drug cycles	Time of biopsy after treatment	AST (IU/L)	ALT (IU/L)	Total bilirubin (mg/dl)	Alkaline phosphatase (IU/L)	Imaging or other lab results	Symptoms	Treatment	Follow-up
1	59/M	Squamous-cell carcinoma of the head and neck	Nivolumab 8	8 months	908	687	0.3	186	CT	Fatigue, abdominal pain	Corticosteroid	Deceased
2	51/M	Pancreatic adenocarcinoma	Nivolumab 5	2 months	134	65	4	310	ERCP, MRCP	Fever, fatigue, abdominal pain, dark urine, pruritis	Corticosteroid	Alive
3	42/M	Melanoma	Nivolumab 2	2 months	198	417	0.9	165	ANA 1:40	Fever, nausea, vomiting	Corticosteroid and cellcept	Alive
4	54/F	Ovarian high-grade serous carcinoma	Pembrolizumab 6	5 months	72	107	0.4	337		Asymptomatic	Corticosteroid	Alive
5	47/M	Melanoma	Pembrolizumab 4	5 months	400	618	0.7	122		Fatigue	Corticosteroid then switched to cellcept	Alive
6	69/M	Melanoma	Nivolumab 1	1 month	437	753	1.2	129		Abdominal pain	Corticosteroid	Alive
7	59/M	Refractory Hodgkin lymphoma	Nivolumab 1	23 days	472	759	5.7	1,100		Fevers abdominal pain, headache	Corticosteroid	Deceased
8	66/F	Glioblastoma	Nivolumab 1	1 month	712	1,999	1.5	106	ANA 1:320	Nausea, headache	Corticosteroid and cellcept	Alive

### Histologic patterns of hepatic toxicity induced by anti-PD-1 therapy

**Figure 1.**
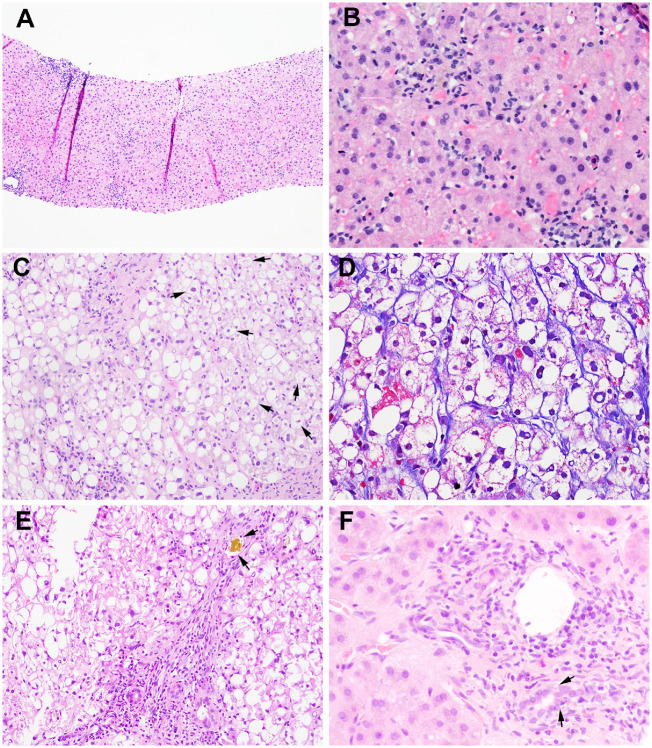
Histologic findings of liver injury induced by anti-PD-1 therapy. (A) and (B) Lobular hepatitis pattern showing lobular inflammation with predominantly lymphocytes, rare plasma cells, eosinophils with acidophilic bodies without significant portal inflammation (case 3, hematoxylin and eosin (H&E) stain; A, 100×; B, 400×); (C) and (D) steatohepatitis pattern showing severe macrovesicular steatosis, many ballooning hepatocytes (arrows), and mild lobular inflammation with perisinusoidal fibrosis (case 2, C, H&E stain, 200×; D, trichrome stain, 400×); (E) besides the steatohepatitic injury pattern, case 2 also showing mild ductal injury and bile plug (arrows) seen in the periportal ductules (H&E, 200×); (F) pure cholestatic-injury pattern showing ductal injury (arrows) present in all of the portal tracts (11/11) and mild portal chronic inflammation (case 7, H&E stain, 400×).

The time interval between the last drug administration and liver biopsy ranged from 23 days to 8 months. The histopathologic findings are summarized in Table 2. Acute lobular hepatitis was detected in six out of eight patients. The lobular inflammation was generally mild and consisted of a mixed inflammatory cell infiltrate with predominantly lymphocytes and rare plasma cells and eosinophils. Lobular spotty necrosis and acidophil bodies were seen in five cases (Figure 1A and B) and centrilobular confluent necrosis was seen in one case. In the case with lobular confluent necrosis, ANA became positive with a titer of 1:320. While a few plasma cells were seen in occasional portal tracts in this case, the portal chronic inflammation was still minimal and no interface hepatitis was identified. Immunohistochemical stains for cytomegalovirus and human herpes simplex types I/II as well as Epstein-Barr Early RNA (EBER) *in* *situ* hybridization were all negative.

**Table 2.** Histologic patterns after anti-PD-1 therapy

Pt#	Portal inflammation	Interface hepatitis	Lobular inflammation	Steatosis	Hepatocyte ballooning	Mallory- Denk bodies	Apoptosis	Bile-duct injury	Ductular reaction	Cholestasis	Fibrosis	Sinusoidal dilatation	Nodular regenerative hyperplasia	Other findings	Histologic pattern
1	(–)	(–)	Mild	(–)	(–)	(–)	(–)	(–)	(–)	(–)	(–)	(–)	(–)	Lobular and perivenular aggregates of macrophages	Lobular hepatitis
2	Mild	Mild	Mild	>90% macrovesicular	Frequent	Rare	(–)	Focal	Mild	(+)	Stage 2	(–)	(–)	Iron: 1+	Steatohepatitis and cholestasis
3	Minimal	(–)	Mild with spotty necrosis	(–)	(–)	(–)	Few	Focal	Minimal	(–)	(–)	(–)	(–)	Microgranulomas; perivenulitis	Lobular hepatitis
4	Minimal	(–)	Centrilobular necrosis	15% macrovesicular	(–)	(–)	Few	(–)	(–)	(–)	(–)	(–)	(–)	Vascular invasion	Lobular hepatitis
5	Mild	(–)	Mild with spotty necrosis	20% macrovesicular	(–)	(–)	Few	(–)	(–)	(–)	(–)	(–)	(–)		Lobular hepatitis
6	Mild	(–)	Mild with spotty necrosis	5% macrovesicular	(–)	(–)	Few	(–)	(–)	(–)	(–)	(–)	(–)		Lobular hepatitis
7	Mild	(–)	Mild	(–)	(–)	(–)	Few	Diffuse	(–)	(+)	(–)	(–)	(–)		Cholestasis
8	Mild	(–)	Centrilobular necrosis	(–)	(–)	(–)	(–)	(–)	(–)	(–)	(–)	(–)	(–)		Lobular hepatitis

No significant inflammation or fibrosis was identified in the portal tracts of these six cases with lobular hepatitis. Three cases showed mild large-droplet macrovesicular steatosis (5%, 15%, and 20%), without histologic evidence of steatohepatitis.

The two remaining cases exhibited different patterns of hepatic injury. One case demonstrated steatohepatitis with severe large-droplet macrovesicular steatosis present in 90% of hepatocytes, frequent ballooned hepatocytes (Figure 1C) with occasional Mallory-Denk bodies, and mild lobular inflammation (NAFLD Activity Score: steatosis 3, lobular inflammation 1, hepatocyte ballooning 2). A trichrome stain showed periportal and ‘chicken wire’ pericellular and perisinusoidal fibrosis (stage 2) (Figure 1D). Focal bile-duct injury and mild mixed canalicular and hepatocellular cholestasis were also observed in this case (Figure 1E). This patient had had a prior liver biopsy due to obesity, which had shown mild steatosis with large-droplet fat present in 15% of the hepatocytes and no ballooned hepatocytes or fibrosis.

The final case showed a cholestatic pattern of injury featuring diffuse bile-duct injury in all of the portal tracts and mixed canalicular and hepatocellular cholestasis (Figure 1F). There was only mild portal and lobular inflammation. No bile-duct loss, periductal fibrosis, periportal cholate stasis, or fibrosis was identified. Immunohistochemical stains for cytomegalovirus and adenovirus were performed and were negative.

### Management and clinical outcomes

Nivolumab or pembrolizumab was discontinued in all cases because of worsening liver function. Additional immunosuppression was also given to all of the patients: five patients received corticosteroids only, two patients received corticosteroids and mycophenolate mofetil (cellcept), and one patient received corticosteroids initially and then was switched to mycophenolate mofetil. All patients had resolution of their symptoms and their transaminases improved significantly. Nivolumab was restarted in two patients, and AST and ALT subsequently rose to a much higher level compared with the level seen during their previous nivolumab treatment. These two patients were subsequently treated with cessation of nivolumab and initiation of corticosteroids, and their abnormal liver functions eventually recovered. After a short period of follow-up (6–8 months), six patients are still alive. One patient died of advanced malignancy and the other patient died of Achromobacter pneumonia and invasive aspergillosis. There were no treatment-related deaths.

## Discussion

In this report, we presented eight patients who were treated with the anti-PD-1 agents nivolumab or pembrolizumab. The clinical presentation, histopathologic findings in the liver, treatment strategies, and outcomes were studied. Multiple patterns of liver injury were observed in these patients, including acute lobular hepatitis, steatohepatitis, and cholestatic injury. Even though a few case reports and small case series have been published to describe liver-biopsy findings of nivolumab-induced liver injury [23, 24], our study provides a larger case series with a more comprehensive analysis of the histopathological features.

The approval of immune checkpoint inhibitors such as anti-PD-1/anti-PD-L1 monoclonal antibodies has dramatically changed the paradigm of cancer therapy. Unfortunately, checkpoint-inhibitor therapy is associated with immune-mediated side effects that result in collateral damage to normal tissues. Checkpoint-inhibitor therapy-induced dermatitis, pneumonitis, colitis, and hepatitis have been reported across several different cancer types. While the toxicities induced by anti-PD-1 therapy alone are rare and generally low-grade, rare severe toxicities, including nivolumab-induced fulminant hepatic failure, have been reported [25–27]. Fulminant and ultimately fatal orthotopic liver-transplant organ rejection was also observed in two pediatric patients with metastatic hepatocellular carcinoma after nivolumab administration [28]. In this study, we described eight cases of hepatic toxicity induced by anti-PD-1 therapy. Even though gastrointestinal symptoms and abnormal liver function were identified in these patients, fulminant hepatic failure was not observed. A consistent pattern of hepatic injury was also not seen histologically.

The most common pattern of hepatic toxicity induced by anti-PD-1 therapy was acute lobular hepatitis with either spotty or centrilobular confluent necrosis (6/8). These findings support results from a recent study in which lobular hepatitis was a predominant pattern of liver injury induced by nivolumab or the anti-CTLA4 agent ipilimumab [24]. The lobular hepatitis in all of the patients was resolved but appeared to happen very slowly, as the inflammation and abundant ceroid-laden macrophages were observed up to 8 months after nivolumab cessation in one case. Interestingly, microgranuloma—a feature that was described previously in an ipilimumab-treated patient [29]—was detected in only one case in our study, suggesting that antitumor granulomatous reactions are uncommon in patients undergoing anti-PD-1 therapy. As shown in Tables 1 and 2, there is no association between the degree of acute lobular hepatitis and the number of cycles of anti-PD-1 therapy. And there is no time-related histologic difference among these cases of different time intervals.

Besides lobular hepatitis, a steatohepatitic pattern of injury was detected in one case in our study. This patient was obese but his liver-function test results were normal prior to nivolumab treatment. Liver biopsy was also performed prior to treatment in this patient and showed only mild macrovesicular steatosis (15%) without histological features of steatohepatitis or significant fibrosis. The patient developed steatohepatitis with stage 2 fibrosis 2 months after five cycles of nivolumab treatment, suggesting that steatohepatitis was truly induced by or associated with nivolumab treatment. Focal bile-duct injury with mild cholestasis was also present after nivolumab treatment in this patient. Interestingly, among cases with lobular hepatitis, mild steatosis was detected in three cases. It is unknown whether the steatosis was induced by anti-PD-1 therapy or was present prior to therapy in these cases.

A pure cholestatic pattern of injury was observed in one case. There was bile-duct injury in all of the portal tracts as well as mixed canalicular and hepatocellular cholestasis in zone 3, with little associated inflammation. No bile-duct loss, cholate stasis, periportal fibrosis, or significant ductular reaction was detected, suggesting that this was an acute cholestatic pattern of injury.

Clinically, there was concern for autoimmune hepatitis in one case with confluent necrosis, as the patient’s ANA titer was within the normal range prior to nivolumab treatment but became positive with a titer of 1:320 after treatment. The patient was treated with corticosteroids and mycophenolate mofetil, and her transaminases improved significantly while on these therapies. However, no typical histologic feature of autoimmune hepatitis was identified in this case. Only a few plasma cells were present in some portal tracts, whereas the portal chronic inflammation was minimal and no interface hepatitis was identified.

Autoimmune hepatitis is often considered in patients receiving anti-PD-1 treatment who develop abnormal liver-function tests because of the known immune-related side effects of these drugs, and this association also explains the frequent treatment of such patients with corticosteroids with or without immunosuppressant drugs. Based on our findings of the liver-injury patterns, no case showed typical features of autoimmune hepatitis, suggesting that corticosteroids plus immunosuppressant drugs may not be necessary in many patients who develop liver dysfunction while on anti-PD-1 therapy.

In summary, we found that nivolumab or pembrolizumab can cause acute lobular hepatitis as well as a cholestatic pattern of injury. Screening patients for abnormal liver-function tests prior to anti-PD-1 treatment and periodic monitoring may help to prevent severe liver injury. Liver biopsies play a key role in identifying the histologic patterns of hepatic toxicity induced by anti-PD-1 therapy. Our findings suggest that, rather than causing classical autoimmune hepatitis, PD-1 inhibitors appear to produce an immune-mediated but nonspecific acute lobular hepatitis. The observations from this study challenge the present recommendation of using corticosteroids/immunosuppressive therapy to treat immune toxicity induced by checkpoint inhibitors. Instead, we suggest that simply stopping the drug may be sufficient, and that steroids and mycophenolate mofetil may not be necessary in many patients.

## Authors’ contributions

D.Z. and J.L. designed and conceived of the study, and finalized the manuscript; J.H., X.D., X.Z., M.F., L.Y., L. A., C. S., X. Z., and X.L. analysed the data and reviewed the manuscript. All authors read and approved the final manuscript.

## Funding

None.

## Conflicts of interest

All authors declare that they have no conflicts of interest.

## References

[1] Postow MA, Callahan MK, Wolchok JD. Immune checkpoint blockade in cancer therapy. J Clin Oncol 2015;33:1974–82. 25605845 10.1200/JCO.2014.59.4358 PMC4980573

[2] Pardoll DM. The blockade of immune checkpoints in cancer immunotherapy. Nat Rev Cancer 2012;12:252–64. 22437870 10.1038/nrc3239 PMC4856023

[3] Dong H, Strome SE, Salomao DR et al Tumor-associated B7-H1 promotes T-cell apoptosis: a potential mechanism of immune evasion. Nat Med 2002;8:793–800. 12091876 10.1038/nm730

[4] Blank C, Brown I, Peterson AC et al PD-L1/B7H-1 inhibits the effector phase of tumor rejection by T cell receptor (TCR) transgenic CD8^+^ T cells. Cancer Res 2004;64:1140–5. 14871849 10.1158/0008-5472.can-03-3259

[5] Fife BT, Pauken KE, Eagar TN et al Interactions between PD-1 and PD-L1 promote tolerance by blocking the TCR-induced stop signal. Nat Immunol 2009;10:1185–92. 19783989 10.1038/ni.1790 PMC2778301

[6] Francisco LM, Salinas VH, Brown KE et al PD-L1 regulates the development, maintenance, and function of induced regulatory T cells. J Exp Med 2009;206:3015–29. 20008522 10.1084/jem.20090847 PMC2806460

[7] Ishida Y, Agata Y, Shibahara K et al Induced expression of PD-1, a novel member of the immunoglobulin gene superfamily, upon programmed cell death. Embo J 1992;11:3887–95. 1396582 10.1002/j.1460-2075.1992.tb05481.x PMC556898

[8] Freeman GJ, Long AJ, Iwai Y et al Engagement of the PD-1 immunoinhibitory receptor by a novel B7 family member leads to negative regulation of lymphocyte activation. J Exp Med 2000;192:1027–34. 11015443 10.1084/jem.192.7.1027 PMC2193311

[9] Brahmer JR, Drake CG, Wollner I et al Phase I study of single-agent anti-programmed death-1 (MDX-1106) in refractory solid tumors: safety, clinical activity, pharmacodynamics, and immunologic correlates. J Clin Oncol 2010;28:3167–75. 20516446 10.1200/JCO.2009.26.7609 PMC4834717

[10] Iwai Y, Ishida M, Tanaka Y et al Involvement of PD-L1 on tumor cells in the escape from host immune system and tumor immunotherapy by PD-L1 blockade. Proc Natl Acad Sci USA 2002;99:12293–7. 12218188 10.1073/pnas.192461099 PMC129438

[11] Robert C, Long GV, Brady B et al Nivolumab in previously untreated melanoma without BRAF mutation. N Engl J Med 2015;372:320–30. 25399552 10.1056/NEJMoa1412082

[12] Topalian SL, Sznol M, McDermott DF et al Survival, durable tumor remission, and long-term safety in patients with advanced melanoma receiving nivolumab. J Clin Oncol 2014;32:1020–30. 24590637 10.1200/JCO.2013.53.0105 PMC4811023

[13] Robert C, Ribas A, Wolchok JD et al Anti-programmed-death-receptor-1 treatment with pembrolizumab in ipilimumab-refractory advanced melanoma: a randomised dose-comparison cohort of a phase 1 trial. Lancet 2014;384:1109–17. 25034862 10.1016/S0140-6736(14)60958-2

[14] Borghaei H, Paz-Ares L, Horn L et al Nivolumab versus docetaxel in advanced nonsquamous non-small-cell lung cancer. N Engl J Med 2015;373:1627–39. 26412456 10.1056/NEJMoa1507643 PMC5705936

[15] Ferris RL, Blumenschein G, Fayette J et al Nivolumab for recurrent squamous-cell carcinoma of the head and neck. N Engl J Med 2016;375:1856–67. 27718784 10.1056/NEJMoa1602252 PMC5564292

[16] Motzer RJ, Rini BI, McDermott DF et al Nivolumab for metastatic renal cell carcinoma: results of a randomized phase II trial. J Clin Oncol 2015;33:1430–7. 25452452 10.1200/JCO.2014.59.0703 PMC4806782

[17] Ansell SM, Lesokhin AM, Borrello I et al PD-1 blockade with nivolumab in relapsed or refractory Hodgkin's lymphoma. N Engl J Med 2015;372:311–9. 25482239 10.1056/NEJMoa1411087 PMC4348009

[18] Overman MJ, McDermott R, Leach JL et al Nivolumab in patients with metastatic DNA mismatch repair-deficient or microsatellite instability-high colorectal cancer (CheckMate 142): an open-label, multicentre, phase 2 study. Lancet Oncol 2017;18:1182–91. 28734759 10.1016/S1470-2045(17)30422-9 PMC6207072

[19] Topalian SL, Hodi FS, Brahmer JR et al Safety, activity, and immune correlates of anti-PD-1 antibody in cancer. N Engl J Med 2012;366:2443–54. 22658127 10.1056/NEJMoa1200690 PMC3544539

[20] Nishino M, Sholl LM, Hodi FS et al Anti-PD-1-related pneumonitis during cancer immunotherapy. N Engl J Med 2015;373:288–90. 26176400 10.1056/NEJMc1505197 PMC4539956

[21] Geukes Foppen MH, Rozeman EA, van Wilpe S et al Immune checkpoint inhibition-related colitis: symptoms, endoscopic features, histology and response to management. ESBO Open 2018;3:e000278. 10.1136/esmoopen-2017-000278 PMC5786923 29387476

[22] McDermott DF, Drake CG, Sznol M et al Survival, durable response, and long-term safety in patients with previously treated advanced renal cell carcinoma receiving nivolumab. J Clin Oncol 2015;33:2013–20. 25800770 10.1200/JCO.2014.58.1041 PMC4517051

[23] Simonelli M, Di Tommaso L, Baretti M et al Pathological characterization of nivolumab-related liver injury in a patient with glioblastoma. Immunotherapy 2016;8:1363–9. 28000537 10.2217/imt-2016-0057

[24] Zen Y, Yeh MM. Hepatotoxicity of immune checkpoint inhibitors: a histology study of seven cases in comparison with autoimmune hepatitis and idiosyncratic drug-induced liver injury. Mod Pathol 2018;31:965–73. 29403081 10.1038/s41379-018-0013-y

[25] Larkin J, Chiarion-Sileni V, Gonzalez R et al Combined nivolumab and ipilimumab or monotherapy in untreated melanoma. N Engl J Med 2015;373:23–34. 26027431 10.1056/NEJMoa1504030 PMC5698905

[26] Robert C, Schachter J, Long GV et al Pembrolizumab versus ipilimumab in advanced melanoma. N Engl J Med 2015;372:2521–32. 25891173 10.1056/NEJMoa1503093

[27] Sarmen S, Tara S. Acute liver failure from anti-PD-1 antibody nivolumab in a patient with metastatic lung squamous cell carcinoma. Austin Oncol 2016;1:1006.

[28] Friend BD, Venick RS, McDiarmid SV et al Fatal orthotopic liver transplant organ rejection induced by a checkpoint inhibitor in two patients with refractory, metastatic hepatocellular carcinoma. Pediatr Blood Cancer 2017;64:e26682. 10.1002/pbc.26682 28643391

[29] Luke JJ, Lezcano C, Hodi FS et al Antitumor granuloma formation by CD4+ T cells in a patient with rapidly progressive melanoma experiencing spiking fevers, neuropathy, and other immune-related toxicity after treatment with ipilimumab. JCO 2015;33:e32–5. 10.1200/JCO.2013.49.7735 PMC4881373 24616309

